# Antitumor components and mechanisms of *Zanthoxylum bungeanum* Maxim with medicine and food homology

**DOI:** 10.3389/fphar.2025.1525313

**Published:** 2025-02-28

**Authors:** Yuhua Du, Shuai Duan, Yi Yang, Joanna Japhet Tibenda, Shicong Huang, Yi Nan, Zhe Zhang, Ling Yuan

**Affiliations:** ^1^ College of Pharmacy, Ningxia Medical University, Yinchuan, China; ^2^ College of Basic Medicine, Ningxia Medical University, Yinchuan, China; ^3^ Key Laboratory of Ningxia Minority Medicine Modernization Ministry of Education, Ningxia Medical University, Yinchuan, China; ^4^ Department of Chinese Medical Gastrointestinal, China-Japan Friendship Hospital, Beijing, China

**Keywords:** *Zanthoxylum bungeanum* Maxim, medicine food homology plant, cancer, anticancer mechanism, anticancer active ingredients

## Abstract

*Zanthoxylum bungeanum* Maxim (*Z. bungeanum*) is a medicinal and edible plant commonly used to improve the flavor of Chinese cuisine due to its unique numbing taste. It is recognized for its medicinal properties, including bodywarming, relieving cold, promoting blood circulation, and alleviating pain. Additionally, *Z. bungeanum* has been extensively studied for its antitumor properties. In this study, various scientific databases and network pharmacology were used to search for information about *Z. bungeanum* and its components for the treatment of tumors. Numerous active components of Z. bungeanum have been identified, demonstrating antitumor properties. We discovered that *Z. bungeanum* can modulate multiple signaling pathways across various targets using network pharmacological predictions, highlighting its strong antitumor potential. The components of *Z. bungeanum* and the traditional Chinese medicine compound containing *Z. bungeanum* can promote apoptosis, arrest the cell cycle, inhibit cell invasion and metastasis, promote autophagy, and increase the sensitivity of chemotherapeutic drugs through P53, PI3K/AKT, Wnt/β-catenin and other signaling pathways, which are effective against various cancers, including hepatocellular cancer, gastric cancer, and breast cancer. *Z. bungeanum* and its extracts have demonstrated promising effects against various tumors, indicating their potential use in future cancer therapies and offering new strategies for tumor treatment. However, clinical studies evaluating the antitumor efficacy and toxicity of *Z. bungeanum* in humans are scarce. Therefore, well-designed clinical trials should be prioritized in the future to establish a solid foundation for its use in cancer treatment.

## 1 Introduction

Cancer is one of the leading causes of human mortality, and its prevention and treatment remain among the most challenging clinical problems (Siegel et al., 2023). With the aging population and poor lifestyle, the incidence of cancer is increasing every year. Currently, the common methods of cancer treatment include surgical intervention, radiotherapy, chemotherapy, targeted therapy, and immunotherapy (Mun et al., 2018). However, the mortality and recurrence rates of cancer remain high, and the side effects of treatment also cause significant pain to patients. This is attributed to the fact that most antitumor drugs kill cancer cells while severely damaging normal cells, making it crucial to find highly effective and low-toxicity drugs to treat and prevent tumor development.

There is a growing interest in medicinal and edible plants that have both culinary and therapeutic properties. Medicinal and edible plants are rich in polysaccharides, proteins, fats, and vitamins (Ma et al., 2022). The concept of replacing pharmacies with kitchens and medicines with food has gained widespread acceptance. Medicinal and edible plants have demonstrated remarkable therapeutic potential for the treatment of various diseases, often exhibiting low toxicity (Qu et al., 2023). Medicinal and edible plants hold significant promise for managing dyslipidemia, offering superior efficacy, acceptability, and commercial value compared to lipid-lowering medications that frequently cause adverse effects (Hu et al., 2022). Various components of edible and medicinal plants exhibit numerous physiological effects, including anti-inflammatory, antiviral, and antioxidant properties (Lu et al., 2022; Wang et al., 2022; Yang et al., 2022; Xiao et al., 2023). Similarly, these active components have demonstrated significant antitumor activity. *Angelica sinensis*, a medicinal and edible plant, can inhibit liver cancer growth (Wang et al., 2017). Hawthorn is a promising candidate for the management of melanoma (Mustapha et al., 2016). The anticancer properties of ginseng have been demonstrated in various types of cancers of the stomach, lungs, liver, colon, and skin (Wargovich, 2001; Cai et al., 2013; Sharma and Goyal, 2015; Yoo et al., 2017; Chen et al., 2021). Consequently, medicinal and edible plants have significant potential in the development of therapeutic agents for treating tumors.

*Z. bungeanum* is also known as Chinese prickly ash or Huajiao in Mandarin. Currently, it is extensively available in China, Korea, Japan, India, and other Asian nations. In 2002, it was officially recognized by the Chinese Ministry of Health as a plant suitable for both medicinal and food applications. It is primarily used as a seasoning in cuisine due to its unique pungency and numbing sensation and its appetite-enhancing effect. *Z. bungeanum* is particularly popular in Sichuan cuisine (Luo et al., 2022). In addition to its culinary applications, *Z. bungeanum* possesses significant medicinal properties. It exhibits detoxifying, hemostatic, analgesic, anti-inflammatory, and antiplasmodial properties (Goodman et al., 2019; Alam et al., 2020; Qi et al., 2024; Wang et al., 2024). Additionally, *Z. bungeanum* benefits the digestive system and is frequently used as a herbal remedy for treating stomach discomfort and relieving physical ailments. Recent research has demonstrated that *Z. bungeanum* and its active components exhibit significant antitumor properties. *Z. bungeanum* extracts have demonstrated efficacy against various cancers, including skin, gastric, and liver cancers. Therefore, the antitumor effects of *Z. bungeanum* and the mechanisms underlying these effects were investigated in this study. We anticipate that developing medicinally active ingredients and related products derived from *Z. bungeanum* for oncological applications may become a significant research hotspot in the future.

**FIGURE 1:**
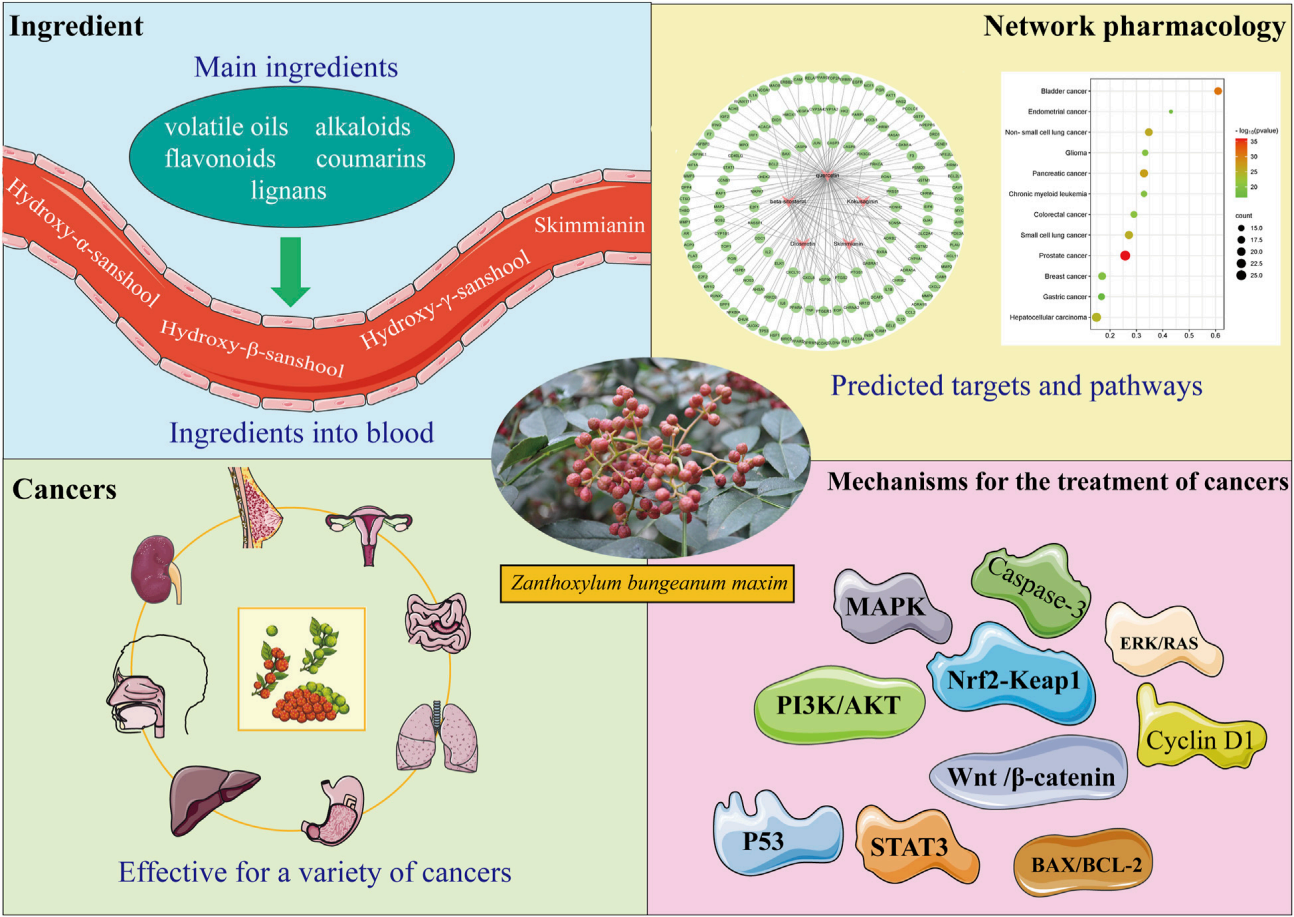
Through literature retrieval and network pharmacology analysis of the components by which *Zanthoxylum bungeanum* Maxim *(Z. bungeanum)* performs its functions, it was discovered that *Z. bungeanum* has curative effects on multiple tumors. Further studies on the antitumor mechanism of *Z. bungeanum* must be conducted to determine the components and mechanisms of its antitumor effects.

First, the composition and blood-entry components of *Z. bungeanum* were investigated using network pharmacological analysis to identify potential components for tumor treatment. Combined with the literature and KEGG analysis, it was discovered that *Z. bungeanum* can fight against various tumors and that *Z. bungeanum* and its constituents inhibit the growth of tumors through multiple target sites and signaling pathways. Additionally, the herbal compounds containing *Z. bungeanum* demonstrated significant antitumor activity, while its hepatoprotective and gastrointestinal protective effects indicate a potential role in preventing tumor development. These findings support the antitumor properties of *Z. bungeanum* and provide new insights into its potential for cancer prevention and treatment (Figure 1).

## 2 Antitumor components of *Z. bungeanum*

### 2.1 Volatile oil

One of the active constituents of *Z. bungeanum* is a volatile oil that contains terpenes, alcohols, and esters. Various early investigations examining the chemical makeup of plants revealed that linalool and limonene were the primary constituents of volatile oil (Yang, 2008; Sun J. et al., 2020).

In Ehrlich ascites tumor model mice, volatile oil demonstrated significant immunomodulatory effects and anticancer efficacy (da Silva et al., 2007). Among the volatile oil components, linalool and limonene have demonstrated promising advantages in the treatment of tumors. Research indicates that linalool exhibits a significant antitumor proliferative effect and lowers the expression of PCNA and Ki-67 in prostate cancer cells 22RV1. These findings demonstrate that linalool can be used as a drug for prostate cancer treatment (Zhao et al., 2020). Colorectal cancer cells undergo apoptosis when exposed to linalool, probably due to cancer-specific hydroxyl radical formation. The linalool group of mice exhibited a 55% lower average tumor weight than the control group (Iwasaki et al., 2016). Additionally, linalool induces cell cycle arrest and stimulates apoptosis by generating oxidative stress and activating MAPK and AKT pathways in hepatocellular carcinoma cells (Rodenak-Kladniew et al., 2018).

D-limonene-induced apoptosis in lung cancer cells was inhibited using the autophagy inhibitor chloroquine and ATG5 knockdown, confirming that D-limonene inhibits tumor growth via the autophagy pathway (Yu et al., 2018). In gastric cancer, D-limonene exhibited anti-angiogenic and pro-apoptotic effects, thereby inhibiting its growth and metastasis (Lu et al., 2004). Besides, D-limonene functions well against numerous cancers, including breast cancer (Mandal et al., 2023), neuroblastoma (Berliocchi et al., 2018), and melanoma (Alipanah et al., 2021).

### 2.2 Alkaloids

Alkaloids are the primary active compounds in *Z. bungeanum*, with over 80 different alkaloids being extracted from this plant (Fu et al., 2020; Ji et al., 2022). Among these, quinoline and isoquinoline alkaloids, including skimmianine, leucine, goitrogenine, and chelerythrine, were the most prevalent. Several studies have reported that alkaloids from the roots of *Z. bungeanum* exhibit cytotoxic and antiproliferative effects against various tumor cell lines, including those from liver, lung, cervical, and stomach cancers (Mbaveng et al., 2019; Fu et al., 2020; Qin et al., 2022). Moreover, the total alkaloids extracted from *Z. bungeanum* root inhibited tumor growth in mice by regulating the blood levels of TNF-α and interleukin-2, as well as by promoting apoptosis. The underlying mechanism of action is likely associated with immune system modulation and induction of tumor cell death (Long et al., 2022).

Furthermore, skimmianine, a major alkaloid in *Z. bungeanum*, induces apoptosis in non-small cell lung cancer (NSCLC) cells, significantly inhibiting their proliferation. The effects on apoptosis and growth inhibition were concentration-dependent and mediated through caspase activation (Zuo et al., 2019). In addition, some scholars have investigated the antitumor activity of chelerythrine from *Z. bungeanum* and discovered that it could reduce p-FAK expression, thereby altering the cytoskeletal structure and inhibiting hepatocellular carcinoma by downregulating MMP-2/9 expression through the PI3K/AKT/mTOR signaling pathway (Zhu et al., 2018).

### 2.3 Flavonoids

*Z. bungeanum* contains a high concentration of flavonoids, predominantly in the form of flavonoid glycosides. The major flavonoid components of *Z. bungeanum* include quercetin, chrysin, rutin, and other active ingredients with anticancer properties (Zhang et al., 2014).

Quercetin is a natural flavonoid component of *Z. bungeanum* that exhibits various activities, including cardiovascular protection, anti-inflammatory, and antitumor activities (Li et al., 2016; Patel et al., 2018; Mirazimi et al., 2022). In the context of antitumor activity, quercetin increases lysosomal activation and ferritin degradation mediated by the transcription factor EB, leading to iron death and P53-independent cell death (Wang et al., 2021). Quercetin inhibits proliferation and metastasis and promotes apoptosis in lung, cervical, and liver cancers (Bishayee et al., 2013; Chang et al., 2017; Ren et al., 2017). Hyperin, extracted from the leaves of *Z. bungeanum*, inhibited the growth of SW620 colon cancer cells through the P53 signaling pathway and caspase-dependent apoptosis (Zhang et al., 2017). Furthermore, hyperin may suppress the progression of gastric cancer by modulating the Wnt/β-catenin signaling pathway (Ping, 2020). Moreover, hyperin glycoside demonstrated good efficacy in the treatment of skin, lung, and ovarian cancers (Zhu et al., 2017; Li et al., 2018; Kong et al., 2020). Rutin, recognized as a safe anticancer agent, exerts its anticancer effects by modulating multiple signaling pathways (Imani et al., 2021).

### 2.4 Other components

Lignans and coumarins from *Z. bungeanum* have demonstrated cytotoxic effects in lung and pancreatic cancer cell lines (Mukhija et al., 2014; Chai and Qiang, 2022). Steroid A, a C34 pentacyclic steroid analog extracted from *Capsicum annuum*, exhibited antiproliferative effects against HeLa, MCF-7, and HepG2 cell lines, exhibiting promising antitumor activity (Meng et al., 2020).

### 2.5 Components entering the blood

Rong et al. discovered that when *Z. bungeanum* extract was injected subcutaneously, the absolute bioavailability of the amide constituents, hydroxy-α-sanshool, hydroxy-β-sanshool, and hydroxy-γ-sanshool, was 100.2%, 76.2%, and 90.3%, respectively (Rong et al., 2016). Lin et al. used UPLC-Q-TOF-MS to determine the alkaloidal constituents in the plasma of rats after oral administration of *Z. bungeanum* extract and detected 18 alkaloids, with skimmianine having the highest maximum plasma drug concentration (377.90 ± 52.65 ng/mL) (Lin et al., 2020). These results indicated that hydroxy-α-sanshool, hydroxy-β-sanshool, hydroxy-γ-sanshool, and skimmianine can be used for medicinal purposes.

Hydroxy-γ-sanshool significantly reduced the mRNA and protein expression levels of Cyclin D1, CDK4, PCNA, P53, P21, Fas, and Caspase 8 in HCT-116 colon cancer cells. Furthermore, Caspase 8 and P53 protein inhibitors significantly reduced the apoptosis and cell cycle arrest induced by hydroxy-γ-sanshool. These findings confirmed that hydroxy-γ-sanshool inhibits tumor growth through P53 and Caspase 8 pathways (Zhaojun et al., 2022).

Skimmianine significantly reduced the growth of xenograft tumors in nude mice, inhibited the proliferation of esophageal squamous cell carcinoma by preventing the activation of ERK1/2, and modulated epithelial-mesenchymal transition (EMT) to limit tumor cell migration and invasion (Liu et al., 2022). Skimmianine induced apoptosis in lung cancer cells and significantly suppressed the growth of four NSCLC cell lines (Zuo et al., 2019). Skimmianine proved to be an active ingredient against tumors.

## 3 Network pharmacology analysis

**FIGURE 2:**
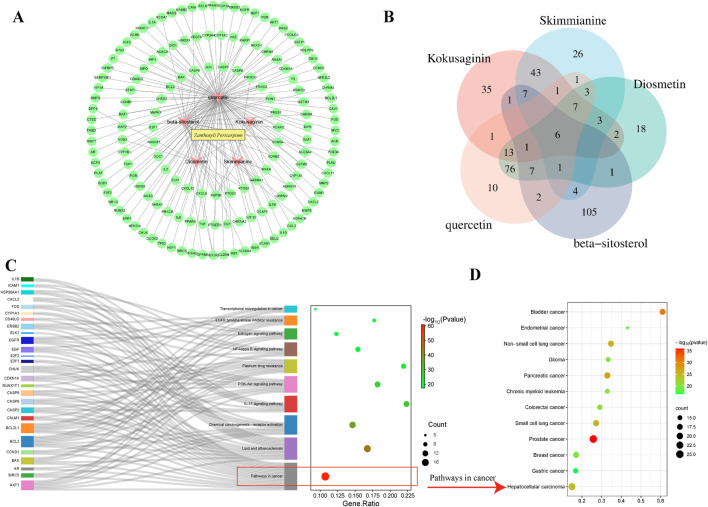
Network pharmacology analysis. **(A)** Components and targets through which *Z. bungeanum* exerts its effects. **(B)** Venn diagram of the targets corresponding to the five components of *Z. bungeanum*. **(C)** Related pathways enriched by the targets of *Z. bungeanum*, among which most targets were enriched in cancer pathways. **(D)** Pathways involved in cancer development.

*Z. bungeanum* was searched in the Traditional Chinese Medicine Database and Analysis Platform database (TCMSP, https://tcmsp-e.com/), according to the oral bioavailability value ≥30% and drug-likeness value ≥0.18. Five active ingredients were selected: kokusaginin, skimmianine, diosmetin, beta-sitosterol, and quercetin. Cytoscape (version 3.8.2) was used to construct the protein-protein interaction network (Figure 2A). The number of targets corresponding to the five components (Figure 2B). KEGG enrichment analysis, conducted using Metascape software (https://metascape.org/), revealed that the targets of *Z. bungeanum* were mainly enriched in cancer pathways (Figure 2C). We further analyzed the cancer pathways and discovered that the targets of *Z. bungeanum* may exhibit a certain effect on bladder, lung, liver, and gastric cancers, among other tumors (Figure 2D).

Among the five active ingredients predicted by network pharmacology, skimmianine, an alkaloid component of *Z. bungeanum*, has been identified as the main component due to its multiple therapeutic effects, including antitumor properties. This validates the network pharmacology predictions and supports the feasibility of further investigating other predicted components for their potential therapeutic benefits. Quercetin, one of the major alkaloidal components of *Z. bungeanum*, has been discussed for its antitumor properties, implying that it can fight cancer.

Kokusaginin significantly increased apoptosis in breast cancer-resistant cells by decreasing P-gp protein levels and inhibiting P-gp function. Kokusaginin has been proposed as an anti-multidrug-resistant drug for the treatment of breast cancer (Chen et al., 2018).

Diosmetin inhibits melanoma tumor metastasis by inducing apoptosis and inhibiting tumor angiogenesis (Choi et al., 2019). Inhibition of RPA2 and RAD51 recruitment at the onset of DNA double-strand breaks during radiotherapy inhibits homologous recombination in endometrial cancer, thereby improving the sensitivity to radiotherapy (Hu et al., 2020). Diosmetin significantly inhibited the proliferation of hepatocellular carcinoma cells and promoted cell cycle arrest in the G2/M phase (Ma and Zhang, 2020). In addition, Diosmetin inhibits the proliferation and metastasis of lung, ovarian, gastric, and colorectal cancers (Koosha et al., 2019; Zhao F. et al., 2021; Song et al., 2022; Zhang and Luo, 2023).

Beta-sitosterol has been demonstrated to reduce the size and scope of tumor metastasis *in vivo*, as well as the proliferation of numerous tumor cell types. Beta-sitosterol promotes apoptosis in breast cancer cells by activating the caspase-8 and Fas receptor pathway proteins (Awad et al., 2007). Additionally, beta-sitosterol is effective against colon cancer, prostate cancer, intracranial aneurysms, ovarian cancer, and fibrosarcoma (von Holtz et al., 1998; Moon et al., 2007; Baskar et al., 2010; Yang et al., 2019; Bae et al., 2021), indicating its potential as an effective compound for preventing and treating tumors.

**FIGURE 3:**
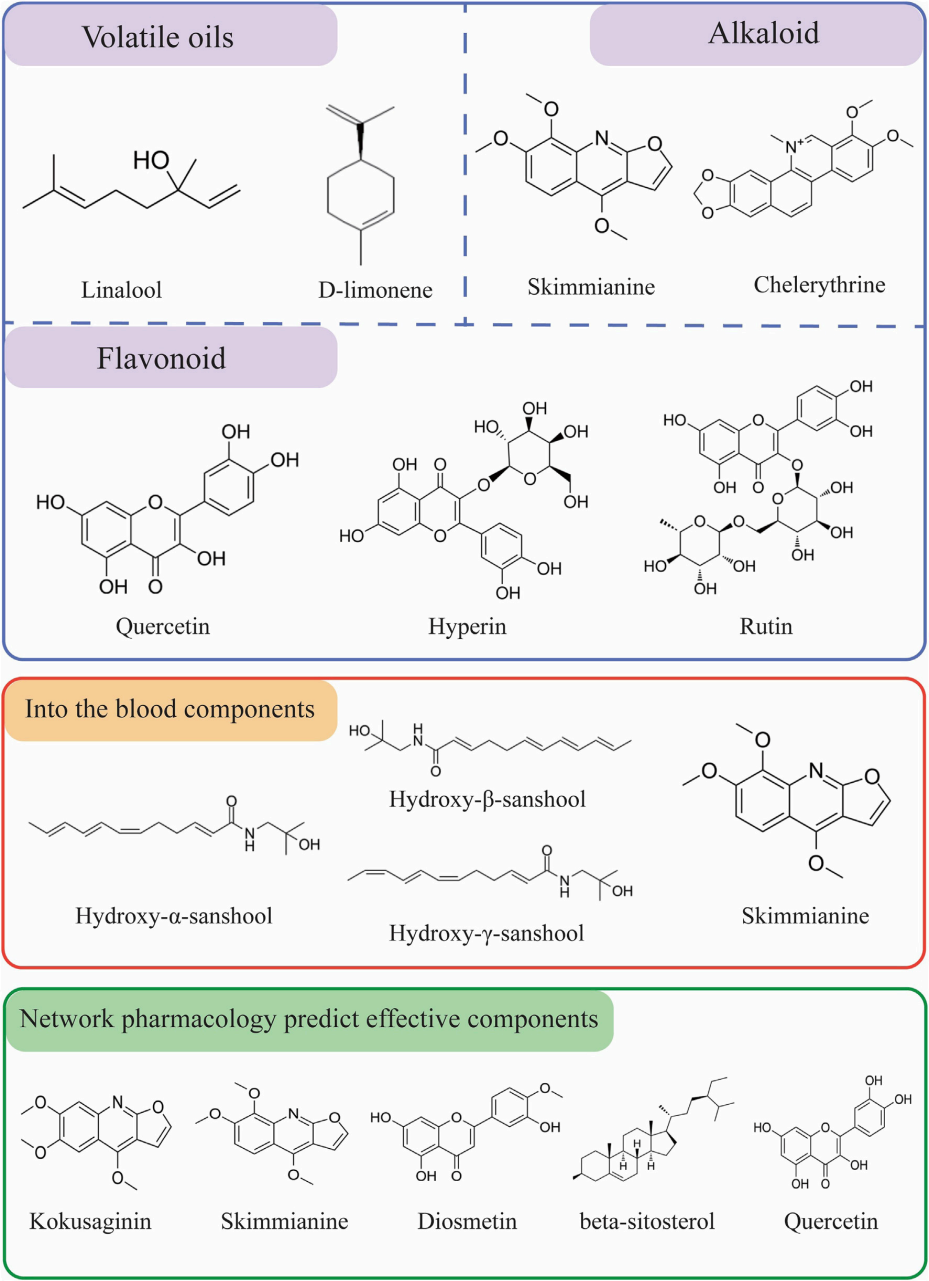
Chemical structures of *Z. bungeanum* components with potential medicinal functions and those entering the bloodstream.

Based on existing studies and web-based pharmacological analyses, we summarized the constituents and blood-entry components of *Z. bungeanum* that may exhibit antitumor properties in Figure 3. The monomers of traditional Chinese medicines are the active ingredients that form the material basis of the mechanism of action. The therapeutic effects of the active ingredients of traditional Chinese medicines on cancer have been widely researched in the medical profession (Li et al., 2020). Identifying the components of *Z. bungeanum* involved in tumor treatment provides a comprehensive understanding of its antitumor mechanisms and a robust foundation for its use as an antitumor agent.

## 4 Antitumor mechanisms of *Z. bungeanum*

### 4.1 Inhibit cell proliferation

The infinite proliferation of tumor cells is the uncontrolled rapid reproduction and growth of cells, producing tumors that are difficult to cure. Inhibition of cell proliferation is an effective strategy to treat tumors, and numerous components of *Z. bungeanum* extract can inhibit the proliferation of tumor cells, thereby achieving the purpose of tumor treatment.

*Z. bungeanum* bark extract inhibited the cellular activity of B16-F10 mouse melanoma cells without exhibiting toxicity to human dermal fibroblasts (Santhanam et al., 2016). Zantholic acid, an active ingredient extracted from *Z. bungeanum,* exhibited cytotoxicity against breast cancer cells, effectively inhibiting cell proliferation (Vyry Wouatsa et al., 2013). Moreover, *Z. bungeanum* leaf extracts exhibited cytotoxicity against promyelocytic and myelomonocytic leukemia cells, further supporting the antiproliferative effects of this medicinal herb (Chou et al., 2011).

### 4.2 Arrest cell cycle

**FIGURE 4:**
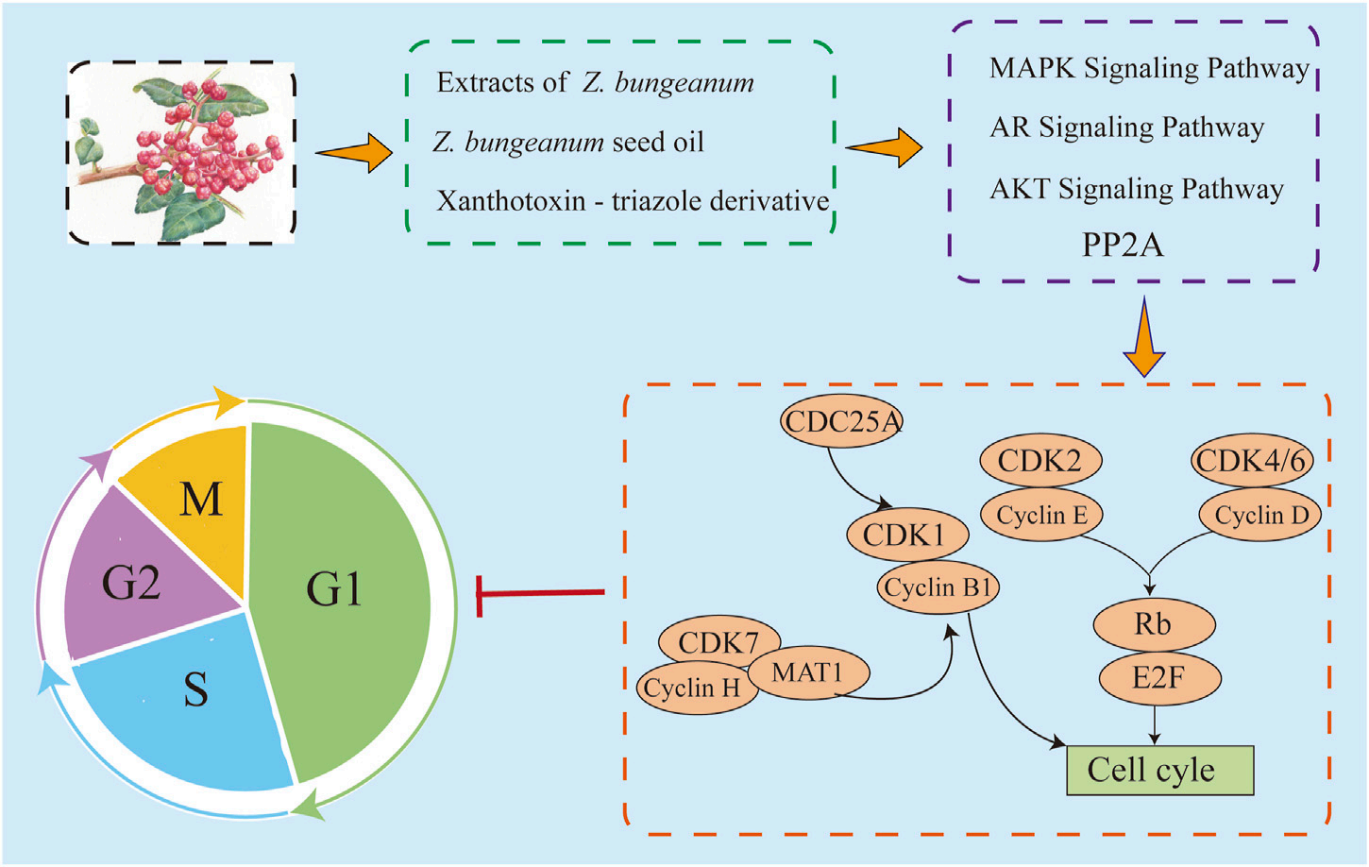
*Z. bungeanum* and its components inhibit cell proliferation by blocking the cell cycle through multiple pathways.

Tumors acquire special abilities during the transition of normal cells into tumor cells, including the ability of tumor cells to proliferate indefinitely due to severe cell cycle dysregulation. The rate of cell proliferation is determined by rhythmic regulation of the cell cycle, which is tightly controlled by various cell cycle-related factors. *Z. bungeanum* extracts may disrupt this regulation by stabilizing specific proteins, thereby impeding cell cycle progression (Hydbring et al., 2016). In hepatocellular carcinoma, *Z. bungeanum* extract significantly inhibited HA22T cell viability in the G2/M phase, which was further confirmed in a nude mouse model, where *Z. bungeanum* extract inhibited tumor growth and activated PP2A proteins to downregulate cell cycle regulatory proteins (Dung et al., 2012). In melanoma, *Z. bungeanum* seed oil inhibited the CDC25A/CyclinB1/CDK1 signaling pathway to block the G0/G1 phase of human malignant melanoma A-375 cells and regulated the MAPK signaling pathway to inhibit cell proliferation, implying that *Z. bungeanum* seed oil exhibits anticancer activity but does not produce toxicity in mice (Pang et al., 2019; Wang et al., 2023). *Z. bungeanum* extract *Z. bungeanum* toxin-triazole derivative induced S/G2 phase arrest in gastric cancer AGS cells by inhibiting cell growth, exhibiting better therapeutic activity and specificity for gastric cancer (Shen et al., 2017). *Z. bungeanum* extract inhibited androgen receptor (AR) signaling and downregulated nuclear levels of AR by inhibiting AKT and Cyclin D1 levels in prostate cancer cells (Yang et al., 2006). The specific mechanism of cell cycle arrest by *Z. bungeanum* and its components is depicted in Figure 4.

### 4.3 Promote cell apoptosis

Apoptosis is a tightly controlled mode of cell death characterized by nuclear consolidation, cellular crumpling, cell membrane vesiculation, and DNA fragmentation. Caspases are cysteine proteases that are crucial for controlling apoptosis (Kopeina et al., 2018). Bax and Bcl-2 are two proteins that play key roles in apoptosis regulation. Their interaction and regulation are essential for the balance between cell survival and death (Hafezi and Rahmani, 2021).

*Z. bungeanum* leaf extract inhibited the activation of the PI3K/AKT pathway and enhanced reactive oxygen species (ROS) production, thereby inducing apoptosis in bladder cancer cells in a dose-dependent manner (Park et al., 2022). *Z. bungeanum* seed oil promoted apoptosis in the laryngeal cancer cell line by inducing autophagy and inhibiting the expression and phosphorylation of PI3K/AKT/mTOR proteins (Bai et al., 2021). *Z. bungeanum* fruit, bark, and leaf extracts, as well as saponins, may exert cytotoxic effects on breast cancer cells through a mechanism involving apoptosis (Vyry Wouatsa et al., 2013; Alam et al., 2017). The decreased expression of IL1β, TGFβ1, and VEGFR1 promotes apoptosis in breast cancer cells (Simanullang et al., 2022).

An alkaloid from *Z. bungeanum*, nitidine chloride, promoted apoptosis in renal cancer cells by inhibiting their proliferation in an effective, time- and dose-dependent manner, thereby inhibiting the growth of renal cancer cells. Moreover, it decreased phosphorylation of AKT and ERK, upregulated BAX, P53, cleavage caspase-3, and cleavage PARP while downregulating the expression of Bcl-2, Caspase-3, and PARP (Fang et al., 2014).

**FIGURE 5:**
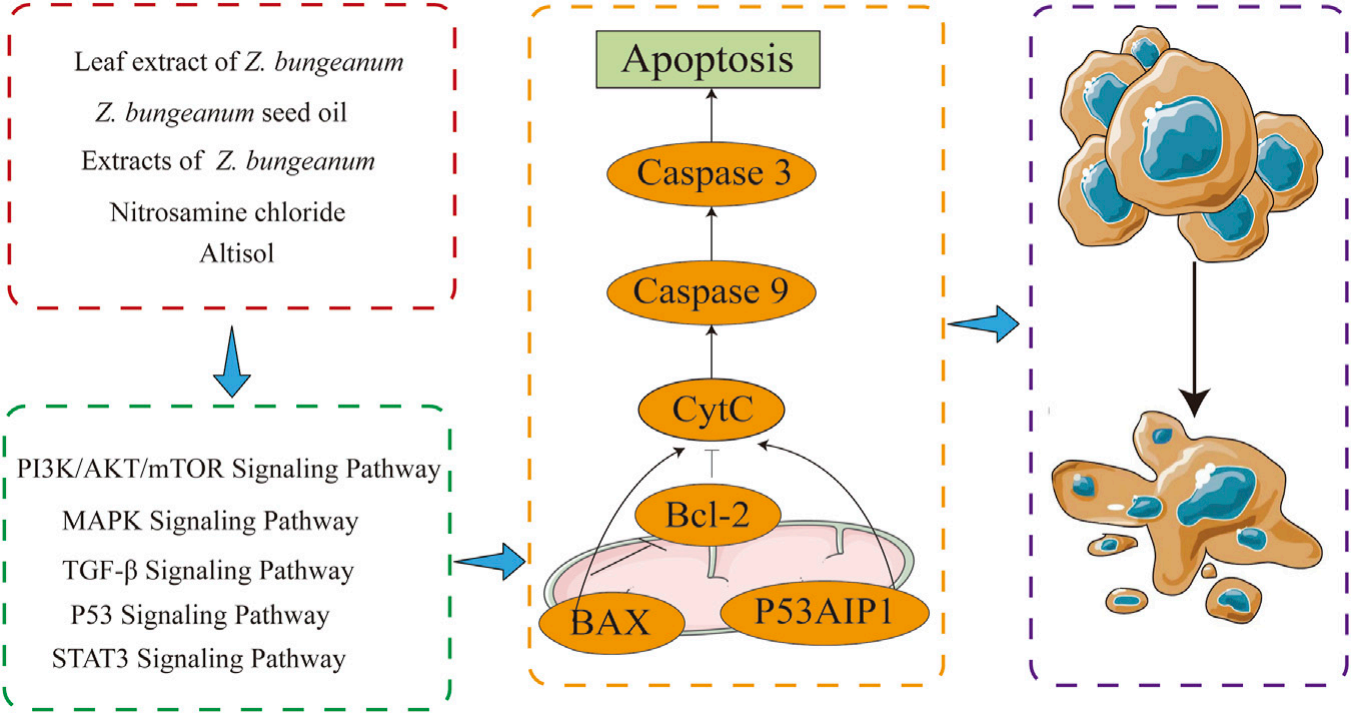
*Z. bungeanum* and its components inhibit cell proliferation by facilitating cell apoptosis through multiple pathways.

In Huh7 hepatocellular cancer cells, ailanthoidol, which was extracted from the bark of *Z. bungeanum,* increased Bax expression and decreased Bcl-xL/Bcl-2 expression. Furthermore, it reduced the expression of mutant P53 protein, thereby preventing STAT3 activation and promoting apoptosis (Tseng et al., 2022). The specific mechanisms by which *Z. bungeanum* and its components promote apoptosis are depicted in Figure 5.

### 4.4 Inhibit cell transfer

Cancer cells invade local tissues and spread to distant sites through invasion and migration, which is the main cause of tumor recurrence. The matrix metalloproteinase family, including MMP1, MMP2, and MMP9, causes cancer cell invasion and migration during cancer progression (Zucker et al., 1994). EMT is a key step in the infiltration and metastasis of tumor cells and is an important marker of malignant tumor progression. Tumor invasion and metastasis are crucial for EMT (Lo and Zhang, 2018).

**FIGURE 6:**
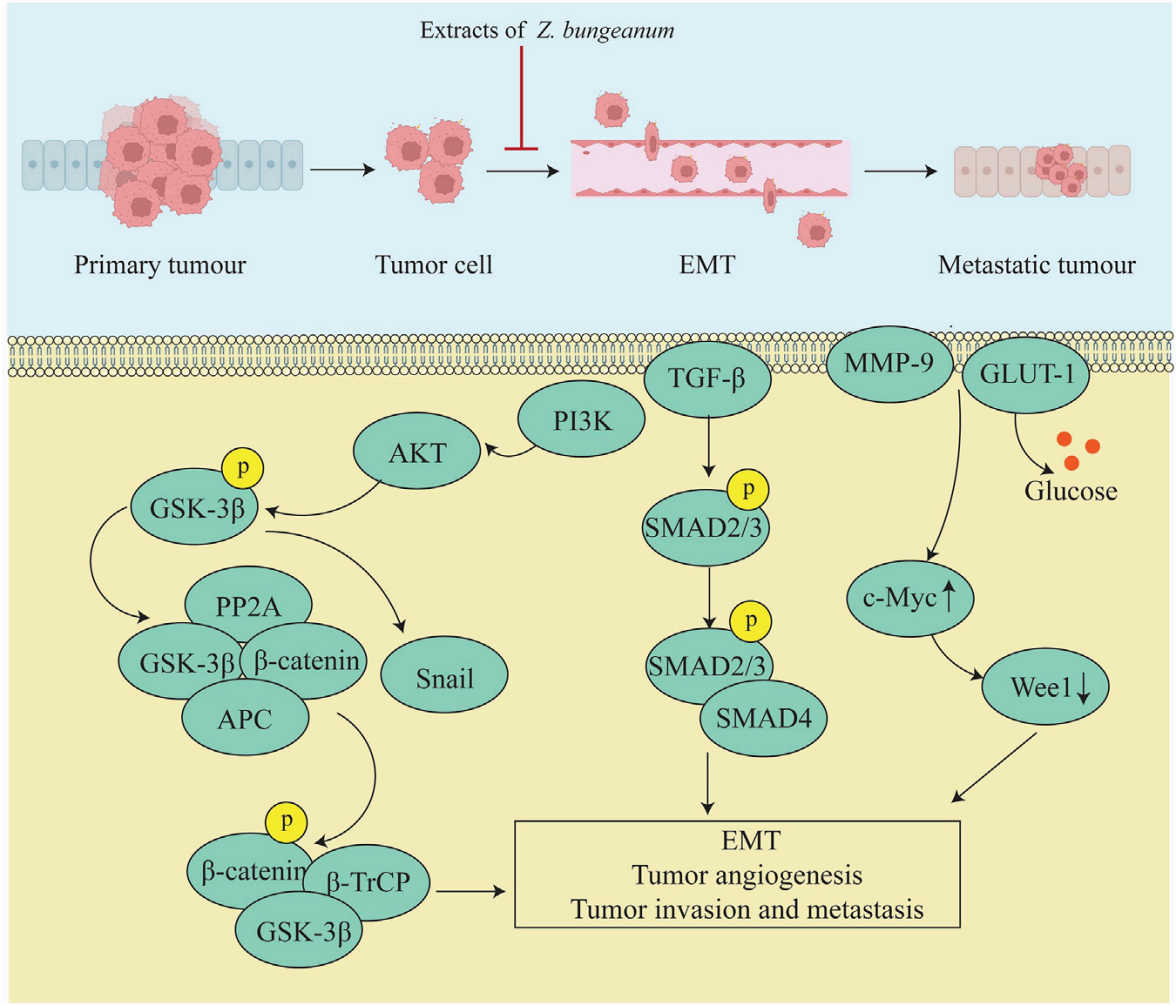
*Z. bungeanum* and its components inhibit tumor metastasis by influencing the EMT process.

Xanthotoxol from *Z. bungeanum* arrests the cell cycle and induces apoptosis and EMT-related genes in NSCLC cells by downregulating PI3K-AKT signaling to inhibit migration and invasion (Lin et al., 2022). *Z. bungeanum* extract enhances GSK-3β and attenuates β-catenin via PP2A, inhibiting the metastasis of HA22T cells and hepatocytes *in vivo* (Dung et al., 2012; Wu et al., 2017). Nitidine chloride, a constituent of *Z. bungeanum*, inhibited EMT and reduced the invasiveness of osteosarcoma cells through the AKT/GSK-3 β/Snail signaling pathway (Cheng et al., 2016). MMP-9 and GLUT-1 enzymes are involved in tumor cell invasion, metastasis, and angiogenesis. *Z. bungeanum* extract can inhibit the metastasis of cervical cancer by reducing the expression of MMP-9 and GLUT-1 in serum and tissues while elevating the expression of Myc and reducing the expression of Wee1 (Ilyas et al., 2022). In addition, quercetin, a component of *Z. bungeanum*, can inhibit the metastasis of pancreatic ductal adenocarcinoma through TGF-β1/Smad2/3 signaling and promote EMT (Guo et al., 2021). The mechanism of inhibition of tumor metastasis by *Z. bungeanum* and its components is depicted in Figure 6.

### 4.5 Increase the sensitivity to drugs

One of the causes of tumor recurrence is the development of resistance in tumor cells. Similar to antibacterial drugs, chemotherapeutic drugs selectively kill non-resistant cancer cells, while the resistant cancer cells frequently reappear, leading to tumor recurrence (Vasan et al., 2019). Consequently, improving the sensitivity of the tumors to drugs is crucial.

**FIGURE 7:**
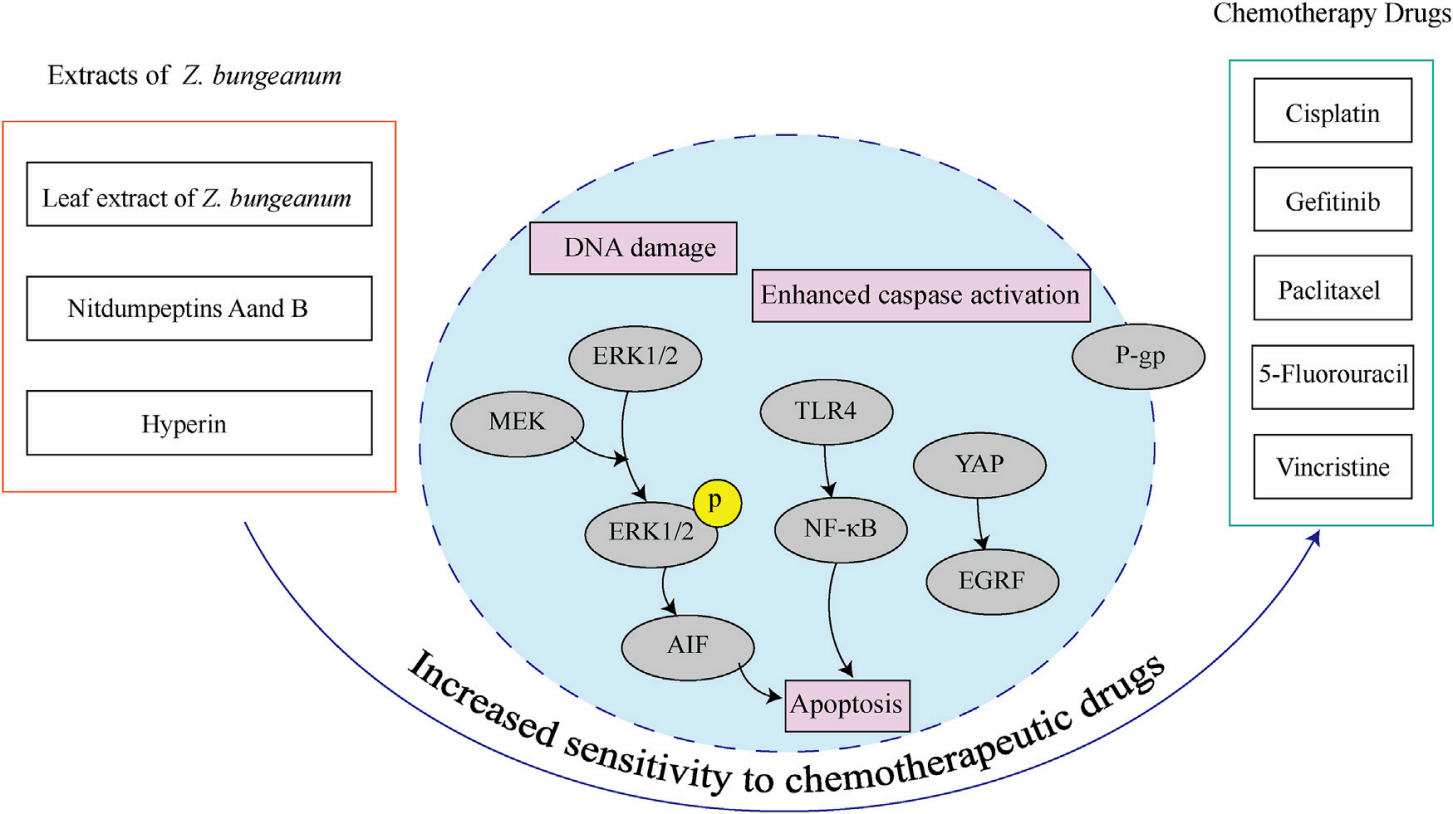
*Z. bungeanum* and its components increase chemotherapeutic drug sensitivity.

In cervical cancer, *Z. bungeanum* leaf extract increased the susceptibility of HeLa cells to cisplatin and other chemotherapeutic drugs by activating the MAPK signaling pathway, which can be used in conjunction with chemotherapeutic drugs to treat cervical cancer (Singh et al., 2015). Nitdumpeptins A and B, cyclic hexapeptides isolated from *Z. bungeanum*, exhibited synergistic antiproliferative effects when combined with gefitinib in gefitinib-resistant NSCLC cells. This combination increases cellular sensitivity to drugs, potentially by suppressing YAP expression in drug-resistant cells (Qin et al., 2021). *Z. bungeanum* contains hyperin, which enhanced the sensitivity of colon cancer cell line HCT8/VCR to vincristine by downregulating P-glycoprotein and inhibiting TLR4 signaling. Besides, hyperin increased the susceptibility of breast cancer cells to paclitaxel (Wang et al., 2018; Sun T. et al., 2020). In conclusion, these results demonstrate that *Z. bungeanum* and its active components could enhance the sensitivity to chemotherapeutic drugs (Figure 7).

### 4.6 Other mechanisms

In cancer cell lines, including HepG2, DLD-1, and Caco-2, *Z. bungeanum* fruit extract increased LC3-II expression, leading to significant autophagy-like cytosolic vesiculation, inhibition of cell division, and, ultimately, induction of cell death (Nozaki et al., 2016). Angoline, extracted from *Z. bungeanum*, was identified as a novel inhibitor of the STAT3 pathway, an important pathway for cancer therapy. Angoline inhibited STAT3 phosphorylation and target gene expression. The identification of small molecules targeting the STAT3 signaling pathway is crucial for the development of novel anticancer therapies (Liu et al., 2014). D-limonene, a key volatile oil constituent of *Z. bungeanum*, can modulate inflammation, oxidative stress, and MAPK pathways, thereby inhibiting skin tumorigenesis in mice (Chaudhary et al., 2012).

**FIGURE 8:**
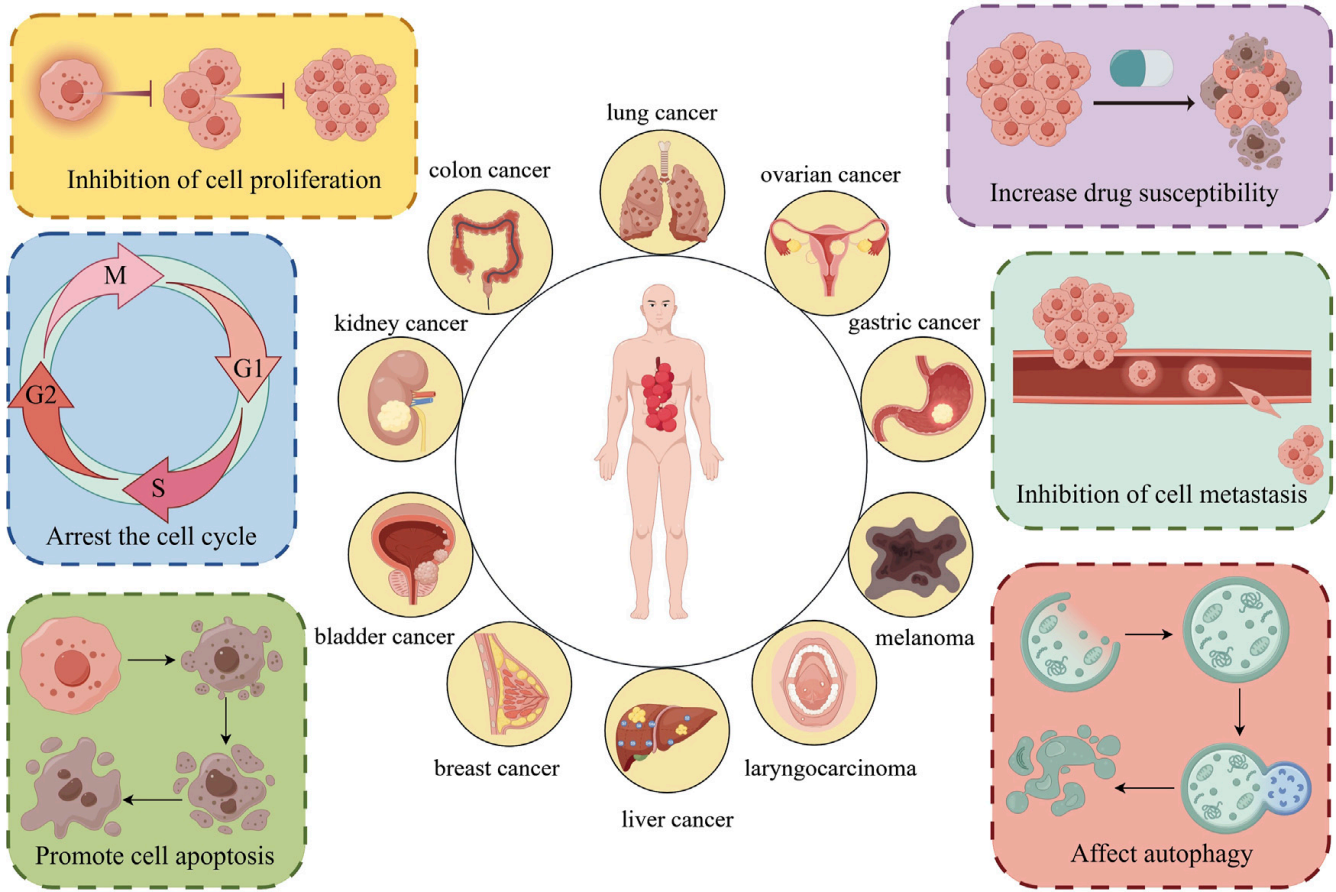
*Z. bungeanum* and its components treat various tumors through multiple mechanisms, providing a basis for their use in tumor therapy. The figure was drawn using Figdraw, http://www.figdraw.com/.

Therefore, we concluded that the antitumor mechanisms of *Z. bungeanum* are complex and diverse; the specific mechanisms are depicted in Figure 8.

## 5 *Z. bungeanum*-related pathways for tumor treatment

We discovered that the antitumor function of *Z. bungeanum* may be achieved through multiple targets and signaling pathways, thereby affecting the mechanisms of tumor cell proliferation, cycle, apoptosis, and metastasis. Studies have demonstrated that the antitumor function of *Z. bungeanum* may be achieved through PI3K/AKT, P53, WNT, STAT3, MAPK, and other signaling pathways. These signaling pathways are closely associated with tumor growth. Table 1 illustrates the precise mechanisms by which *Z. bungeanum* functions as an antitumor agent.

**TABLE 1:** Z. *bungeanum*-related pathways for tumor treatment.

Signal pathway	Ingredients	Cancers	Cells	Mechanics	Literature
PI3K/AKT signaling pathway	Chelerythrine	Liver cancer	Hep3B cells	Inhibition of migration and invasion	Zhu et al. (2018)
	*Z. bungeanum* leaf extract	Bladder cancer	T24 cells	Promotes apoptosis	Park et al. (2022)
	*Z. bungeanum* seed oil	Laryngeal career	Hep-2cells	Promotes apoptosis	Bai et al. (2021)
Wnt signaling pathway	Hyperin	Gastric cancer	AGS, MKN-45cells	Inhibits cell proliferation, migration, and invasion and induces apoptosis	Ping (2020)
P53 signaling pathway	Ailanthoidol	Liver cancer	Huh7 cells	Blocking the cell cycle and promoting apoptosis	Tseng et al. (2022)
	Hyperin	Colon cancer	SW620 cells	Promotes apoptosis and inhibits cell growth	Zhang et al. (2017)
	Hydroxy-γ-sanshool	Colon cancer	HCT-116 cells	Blocking the cell cycle and promoting apoptosis	Zhaojun et al. (2022)
STAT3 signaling pathway	Angoline	Liver cancer	HepG2 cells	Inhibits STAT3 and cell proliferation	Liu et al. (2014)
MAPK signaling pathway	Skimmianine	Esophageal Cancer	TE-1, Eca109 cells	Inhibits cell proliferation and regulates EMT to block tumor cell migration and invasion	Liu et al. (2022)
	*Z. bungeanum* leaf extract	Cervical cancer	Hela cells	Increased drug sensitivity to chemotherapeutic agents	Singh et al. (2015)
	*Z. bungeanum* seed oil	Melanoma	A-375 cells	Regulates cell cycle and promotes apoptosis	Pang et al. (2019)
AR signaling pathway	*Z. bungeanum* extract	Prostatic cancer	LNCaP cells	Regulates cell cycle and promotes apoptosis	Yang et al. (2006)
Nrf2/keap1 signaling pathway	*Z. bungeanum* extract	Breast cancer	Breast cancer in rats	Antioxidant effect	Sahu et al. (2021)
Autophagy signaling pathways	D- limonene	Lung cancer	A549, H1299 cells	Promotes autophagy and inhibits cell proliferation	Yu et al. (2018)
	*Z. bungeanum* fruit extract	Variety of tumor	DLD-1, HepG2, Caco-2cells	Promotes autophagy and inhibits cell proliferation	Nozaki et al. (2016)

## 6 Anticancer effect of traditional Chinese medicine compound of *Z. bungeanum* to treat tumors

*Z. bungeanum* has been part of the Chinese Pharmacopoeia since 1977. It is included in over 30 types of prescription medications used to treat numerous illnesses, including dermatitis, dyspepsia, vomiting, diarrhea, and abdominal discomfort. Dajianzhong decoction and Wumei pill are the most widely used. In modern research, numerous Chinese herbal compound prescriptions containing *Z. bungeanum* have been demonstrated to be effective in treating tumors.

Dajianzhong decoction can inhibit gastric cancer proliferation and metastasis by regulating MMP-9 expression by modulating the ERK1/2 signaling pathway (He et al., 2017). Wumei pill suppressed lung cancer progression by inhibiting the HGF/C-Met signaling pathway (Yu et al., 2022). Moreover, it significantly inhibited the proliferation, migration, and invasion of pancreatic cancer and induced apoptosis by inhibiting the PI3K/AKT signaling pathway (Wang and Zhang, 2022). Furthermore, Wumei pill inhibited the development of gastric cancer and precancerous lesions and significantly downregulated the expression of c-myc and surviving genes (Li et al., 2010). Xingma Biejia decoction can improve the survival status and survival time of mice with acute myeloid leukemia, as well as the histopathological damage of the liver, spleen, and bone marrow. This could be due to controlling the rate at which mitochondria divide and triggering cellular autophagy (Si et al., 2023).

In conclusion, it has been proven that the compound formula containing *Z. bungeanum* can treat certain tumors, in which *Z. bungeanum* plays an indispensable role. Plasma and urine analyses of people who had taken Dajianzhong decoction revealed that hydroxy-α-sanshool and hydroxy-β-sanshool were present in plasma, with maximum values of these two compounds appearing at 0.5 h after drug administration. Additionally, glucuronic acid conjugates of these two chemical compounds were detected in the urine, confirming the role of *Z. bungeanum* in antitumor therapy (Iwabu et al., 2010; Jiang et al., 2023).

## 7 *Z. bungeanum* for tumor prevention

Tumor development is a long process, and timely intervention before cancer onset can significantly reduce tumor occurrence. Early intervention can effectively lower the cancer risk, facilitate preventive measures, and promote overall health maintenance. Such strategies play a crucial role in reducing the incidence of tumors and mortality rates (Vineis and Wild, 2014).

In terms of liver protection, *Z. bungeanum* alkaloids improved liver and kidney function markers in olive oil-induced liver cancer (Acheampong et al., 2021). Glycoproteins isolated from *Z. bungeanum* fruits can act as potent hepatoprotective agents via the antioxidant pathway (Lee and Lim, 2008). *Z. bungeanum* and its components improve non-alcoholic fatty liver disease by regulating fatty acid and cholesterol metabolism, intestinal flora, and activating the AMPK/Nrf2 signaling pathway (Huang et al., 2023; Peng et al., 2024). These studies indicate that *Z. bungeanum* might be effective in preventing liver cancer.

**FIGURE 9:**
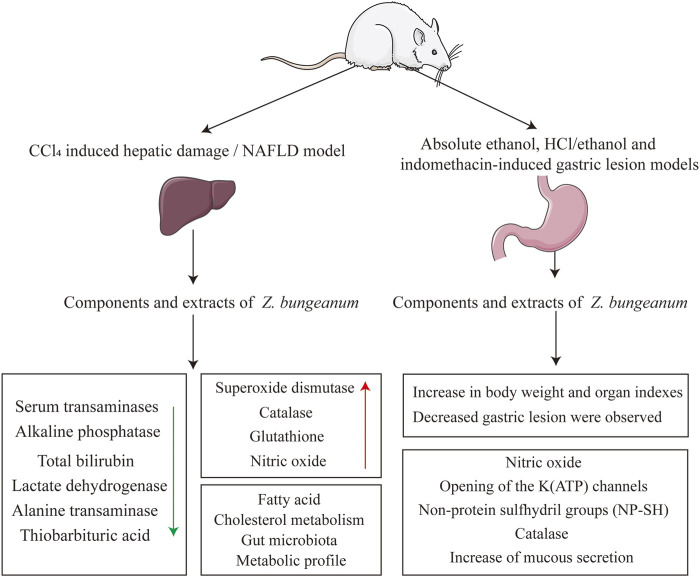
Mechanism of *Z. bungeanum* preventing tumor: Liver and stomach function protection.

The gastroprotective function of *Z. bungeanum* has been confirmed by several studies. Unsaturated fatty acid amides isolated from *Z. bungeanum* pericarp can relax the circular muscles of isolated guinea pig stomachs and contract the longitudinal muscles of the ileum and distal colon, thereby regulating gastrointestinal motility (Hashimoto et al., 2001). *Z. bungeanum* stem bark extract has also demonstrated significant gastroprotective effects (Freitas et al., 2011). *Z. bungeanum* pericarp extract increased the body weight and decreased gastric lesions in rats. *Z. bungeanum* and its components play a role in tumor prevention by protecting the liver and stomach (Figure 9).

## 8 Conclusion and prospects

The uncontrolled proliferation and metastasis of tumors make their treatment difficult, while resistance to chemotherapeutic drugs and the off-target effects on normal cells hinder their complete eradication. This study highlighted the substantial role of medicinal and edible plants in cancer therapy by increasing the sensitivity of tumor cells to chemotherapy with minimal toxicity to normal cells. Therefore, medicinal and edible plants can be used as treatments for cancer or as adjuvant therapies to conventional treatments, helping alleviate the burden on cancer patients.

The rind of *Z. bungeanum* possesses a strong hemp flavor and serves as an important seasoning, primarily due to its high volatile oil content, making it a valuable raw material for spices and flavoring agents. Additionally, *Z. bungeanum* leaves can be used in stir-fries, cold dishes, and tea preparations. Recently, *Z. bungeanum* has been increasingly used in healthcare products, including foot bath packs, foot patches, and teas, where it has demonstrated antibacterial, insecticidal, analgesic, and cold-repelling properties. Additionally, *Z. bungeanum* is commonly used in pest control for the storage of archives, food, clothing, and Chinese medicinal tablets. The versatility of this plant in both medicinal and culinary applications highlights its significance across multiple domains.

This review provides a systematic account of the antitumor properties of *Z. bungeanum*, providing solid theoretical support for researchers investigating its anticancer potential. The antitumor properties of *Z. bungeanum* were thoroughly explored and analyzed using network pharmacology. Previous studies have confirmed that *Z. bungeanum* extract exhibits therapeutic effects on various tumors, including hepatocellular, gastric, and lung carcinomas. These effects are mediated through the modulation of multiple signaling pathways, including P53, WNT, and PI3K/AKT, leading to the inhibition of cell proliferation, suppression of cell migration and invasion, and enhancement of the sensitivity to chemotherapeutic agents, among other multifaceted mechanisms.

Avicularin, a flavonoid component of *Z. bungeanum* peel, can inhibit ferroptosis and improve cognitive impairment in Alzheimer’s disease by regulating the NOX4/Nrf2 axis (Li et al., 2024). WGX50 from *Z. bungeanum* alleviated doxorubicin-induced cardiotoxicity by inhibiting mitochondrial ROS and ferroptosis (Tai et al., 2023), demonstrating that the components of *Z. bungeanum* play a regulatory role in ferroptosis. Moreover, ethyl acetate extract of *Z. bungeanum* can improve cognitive dysfunction in aged mice by inhibiting NLRP3 inflammasome activation and pyroptosis (Zhao M. et al., 2021). Compounds isolated from *Z. bungeanum* inhibited LPS-induced nitric oxide production in RAW264.7 cells (Liu et al., 2023), indicating that *Z. bungeanum* affects immune function. *Z. bungeanum* polysaccharides also exhibit antioxidant activity (Liu et al., 2024). However, there are no relevant reports on the effect of Z. bungeanum on tumor growth by promoting ferroptosis and pyroptosis. More research is required to elucidate the role of Z. bungeanum in tumor treatment. The antitumor properties of *Z. bungeanum* have not been comprehensively investigated, and there is still a partial lack of understanding of the mechanism, thereby requiring in-depth research to investigate the antitumor potential of *Z. bungeanum* thoroughly.

The anticancer potential of *Z. bungeanum* has yet to be conclusively established through extensive rigorously designed clinical trials. While network pharmacology, as well as *in vivo* and *ex vivo* studies, have demonstrated its potential as an adjuvant therapy in cancer treatment, further validation through clinical trial data is necessary due to the complexity of integrating traditional Chinese medicine into cancer therapy. We anticipate that *Z. bungeanum* and its active ingredients can be applied in clinical practice to treat tumors, offering new therapeutic options for cancer patients. Our research aimed to take advantage of the low toxicity and high efficacy of *Z. bungeanum* to address the current challenges in cancer therapy and alleviate treatment-related side effects in patients.

## Glossary

Z. bungeanum: *Zanthoxylum bungeanum* Maxim
P53: Cellular tumor antigen p53
PI3K: Phosphoinositide 3-kinase
AKT: Protein kinase B
Wnt: Wingless/integrated
UPLC: Ultra Performance Liquid Chromatography
MS: Mass spectrum
KEGG: Kyoto Encyclopedia of Genes and Genomes
OB: Oral bioavailability
DL: Drug-like properties
PCNA: Proliferating Cell Nuclear Antigen
Ki-67: Protein phosphatase 1
MAPK: Mitogen-activated protein kinase
ATG5: Autophagy-related Gene 5
TNF-α: Tumor necrosis factor-α
IL-2: Interleukin-2
NSCLC: Non-small cell lung cancer
FAK: Focal adhesion kinase
mTOR: Mammalian target of rapamycin
MMP2/9: Matrix metalloproteinase 2/9
CDK4: Cyclin-dependent kinase 4
HGF: Hepatocyte growth factor
ERK1/2: Extracellular signal-regulated kinase
EMT: Epithelial-mesenchymal transition
ROS: Reactive oxygen species
P-gp: P-glycoprotein
BAX: BCL-2-associated X protein
BCL-2: B cell lymphoma-2
GLUT-1: Facilitative glucose transporter-1
**PA**RP: Poly ADP-ribose polymerase
STAT3: Signal transducer and activator of transcription 3
LC3-II: Microtubule-associated protein 1 light chain 3 II
MYC: Myc proto-oncogene protein
CDC25A: M-phase inducer phosphatase 1
Nrf2: Nuclear factor erythroid2-related factor 2
GSK-3β: Glycogen Synthase Kinase 3β
TLR4: Toll-like receptor 4
YAP: Yes-associated protein
TGFβ1: Transforming growth factor beta 1
